# Is Balance Control Affected by Sleep Deprivation? A Systematic Review of the Impact of Sleep on the Control of Balance

**DOI:** 10.3389/fnins.2022.779086

**Published:** 2022-05-16

**Authors:** Guilherme Silva Umemura, Fabianne Furtado, Fabia Camile dos Santos, Bruno da Silva Brandão Gonçalves, Arturo Forner-Cordero

**Affiliations:** ^1^Biomechatronics Laboratory, Department of Mechatronics, Escola Politécnica, University of São Paulo, São Paulo, Brazil; ^2^Federal Institute of Education, Science and Technology of Southeast of Minas Gerais, Barbacena, Brazil; ^3^Department of Psychiatry, Federal University of São Paulo, São Paulo, Brazil

**Keywords:** actigraphy, actimetry, postural control, posture, sleep deprivation (SD), circadian rhythm

## Abstract

**Background:**

Sleep is a complex physiological function that should be addressed from different perspectives and consider the circadian rhythm. Sleep deprivation, either acute or chronic, negatively affects several functions, including motor control. Balance control is essential in several daily life activities and balance problems are related to falls.

**Research Question:**

This review focuses on how sleep conditions impact balance control.

**Methods:**

Systematic literature review according to PRISMA guidelines.

**Results:**

The literature provided strong evidence that acute sleep deprivation impairs postural control. Chronic sleep deprivation as well as low sleep quality had similar effects, although there is a lower number of works addressing this issue. Furthermore, time awake worsens postural controls and it can be used to detect sleepiness and fatigue. The sleep deprivation showed a stronger negative effect on postural control when removing the visual information (eyes closed) than when reducing proprioceptive feedback (soft surface). There is scarce literature about the effects of chronotype, circadian patterns and chronic sleep deprivation, a frequent problem, on balance control; however they consistently indicate that there is an relationship between them. Most of the studies only consider one-night (acute) sleep deprivation without monitoring prior sleep conditions and the circadian rhythm phase of the participants. However, a few studies indicated that these factors must be considered.

**Significance:**

These results suggest that the sleep conditions of a subject should be considered for several days prior to balance control tests. Therefore, we propose a revision of current postural measurement protocols to include sleep assessment, such as sleep quality questionnaires or actimetry, and to consider the circadian rhythm of the participants to plan the hour of the tests.

## Introduction

The current lifestyle seems to compel people to sleep less than they would like to and this behavior has originated a largely neglected epidemic of chronic sleep deprivation (Bonnet and Arand, 1995). Sleep is a physiological function essential for cognitive and motor performance. Sleep disturbances affect learning, memory as well as attention and cognition in multiple ways (McCoy and Strecker, 2011). The effect of sleep disturbances on motor performance, accuracy, speed, learning and balance control has been studied in the last decades (McCoy and Strecker, 2011; Al-Sharman and Siengsukon, 2013). However, so far, there has been no review about the effects of sleep deprivation on balance. It is very important to study the influence of sleep deprivation on motor performance due to the increased risk of accidents, work-related injuries, and, in the case of balance, possible causes of falls in frail populations, such as the elderly or patients with balance disorders (Alvarez and Ayas, 2004; Czeisler et al., 2016).

The sleep-wake cycle is regulated by a circadian/homeostatic process (Daan et al., 1984). In the homeostatic regulation process, the sleep pressure increases exponentially between the beginning of wakefulness and the beginning of sleep. From this instant, it starts recovering with a decreasing exponential function. Sleep homeostasis also acts with the circadian process, although these two processes perform independent functions. Sleep homeostasis regulates the structure and timing of sleep, while the circadian process adjusts synchronization processes and regulates brain functions regardless of bedtime (Santhi et al., 2016). Both processes, with large individual variations, result in cognitive oscillations that affect the performance of daily tasks (Wyatt et al., 1999).

From those large individual variations it is possible to classify subjects in chronotypes, which reflects how the individual circadian oscillator is synchronized within the natural light/dark cycle (Roenneberg et al., 2007): morning people prefer to perform their tasks at earlier times, and afternoon people prefer to perform their tasks at later times (Horne and Ostberg, 1976; Wittmann et al., 2006). The first approach to classify the subjects' chronotypes were proposed in the Morningness and Eveningness Questionnaire (MEQ-HO) (Horne and Ostberg, 1976). It was based on questions about ideal situations for optimal performance of daily tasks. Furthermore, another questionnaire, based on sleeping and waking times during working and free days, aims at classifying individuals into different groups, with the addition that it is possible to assess the sleep restriction caused by working days, a condition known as social jetlag (Wittmann et al., 2006).

There are many types of sleep disturbances related to different types of pathologies. However, sleep in healthy people can be affected by a lack of quantity and/or quality. Sleep deprivation (SD) describes a state caused by inadequate sleep quantity or quality. This restriction of sleep to below the needs of the subject can be acute or chronic. Acute or short-term sleep deprivation is a total or severe reduction in the usual sleep time for 1 or 2 days (Koslowsky and Babkoff, 1992), as it is common in military or clinical personnel.

Chronic sleep deprivation is the partial sleep restriction that accumulates for several days, weeks or even months, generating a sleep debt. It occurs when an individual routinely sleeps with insufficient sleep quality and/or less time than needed, depending on the age, less than 7 h per night (Basner et al., 2013; Hirshkowitz et al., 2015). This is the most prevalent condition among the general population. One in three Americans sleeps less than seven hours per night (McCoy and Strecker, 2011), reporting more daytime sleepiness than Europeans (Ohayon et al., 2002; Ohayon, 2010). The Institute of Medicine (USA) estimates that 50–70 million adults suffer from chronic sleep disorders (Colten and Altevogt, 2006).

However, chronic sleep deprivation can occur in different ways. The most obvious one is the reduction in the absolute sleep time, as previously mentioned. It is relatively common to have sleep fragmented by brief arousals, thus impairing the sequence of sleep stages and decreasing sleep quality. Less prevalent is the selective sleep stage deprivation in which one specific sleep stage is missing or reduced, causing a sleep quality reduction. Therefore, there are several sleep disorders that affect sleep quality and may impact balance control performance (Furtado et al., 2016).

Several studies have been published about how sleep deprivation affects balance control (Frey et al., 2004; Forsman et al., 2013; Zouabi et al., 2016). However, the mechanisms that relate SD with motor performance are not fully understood and there are divergent results in the literature. These differences may be due to methodological differences between tests that should control factors related to different fields of knowledge, such as sleep, biomechanics and motor control. In this respect, aspects related to sleep, such as hours of SD, time of the experiment or sleep monitoring on days prior to the test, are not usually considered in biomechanics and balance control studies (Duarte and Freitas, 2010). Moreover, the experimental assessment of balance involves considering multiple methodological issues that could influence the results, such as sampling frequency, test duration, type of perturbations applied and balance control parameters chosen (Raymakers et al., 2005; Duarte and Freitas, 2010).

Postural control describes the task of controlling the body position in space for stability, guaranteeing balance and orientation of the body segments for perception and action. In this respect, stability recovery and anticipatory postural adjustments are functions of the posture control system crucial for daily life activities. The task of postural control for balance, or balance control, involves controlling the body position either in a fixed posture, also named static balance, such as in quiet stance, or altering the body configuration in a dynamic balance task, such as standing on a moving platform (Shumway-Cook and Woollacott, 1995; Woollacott and Shumway-Cook, 2002). Postural control requires the integration of different sensory systems, that is, weighting the visual, vestibular and proprioceptive sensory systems to produce adequate muscle tonus and motor actions to maintain balance (Horak, 2006; Ivanenko and Gurfinkel, 2018).

Posturography consists of a set of techniques to measure posture and assess the systems involved in balance: visual, vestibular and somatosensory (Raymakers et al., 2005; Duarte and Freitas, 2010). A force platform, widely used to assess balance, registers the three-dimensional ground reaction forces under the feet. The Center of Pressure (COP) can be calculated as the point of application of the resultant of these ground reaction forces. The COP provides a measurement of postural sway variation with time. Other tools used to assess balance include motion measurement systems based on cameras or inertial sensors. Various parameters, including mean, maximum and minimum sway amplitude, peak-to-peak amplitude, sway path and velocity, root-mean-square (RMS) amplitude and velocity, have been derived from COP data to quantify balance alterations (Raymakers et al., 2005).

Several studies have shown a decreased performance in the control of balance after one night of sleep deprivation. In addition, more recent studies pointed out that balance control can be impaired even under more subtle sleep restriction conditions, such as chronic sleep deprivation or social jetlag, that is, the difference in sleeping times between working and free days. In this context, we aim at finding evidence to decide if posture assessment must or not consider the sleep conditions of the participants and provide guidelines to consider the relation between sleep and balance control performance. Thus, the goal of this literature review is to determine the effects of sleep deprivation on balance control in healthy individuals. There are two main applications of this work. First, it can be relevant to include an evaluation of the sleep state and circadian rhythm of the subject before balance tests to eliminate confounding factors. Second, balance tests could also provide a procedure to assess sleep conditions in populations such as shift workers.

## Methods

### Study Design and Search Strategy

The literature review followed the PRISMA guidelines (Moher et al., 2009) and only peer-reviewed papers were considered. It was registered since 2016 in the PROSPERO International prospective register of systematic reviews under number CRD42016032859.

The search was performed across relevant databases until July 2019: Pubmed/MEDLINE, CINAHL, COCHRANE, Academic Search Premier, Web of Science, SPORTDiscus, and SCOPUS. The search included all the articles containing the term “Sleep Deprivation” in combination with any of the terms: “Postural Balance”, “Posturography”, “Postural Control”, “Postural Sway”.

### Eligibility Criteria and Selection Procedure

Two authors (AFC and FF) performed the searches independently and compared them. Afterward, the articles were screened according to the following eligibility criteria:

- healthy adults, not pregnant;
- a clear indication of sleep deprivation and;
- clearly presenting balance control measurements registered before and/or during and/or after a sleep deprivation period.

No restriction was applied regarding language, publication year, sex, and types of sleep deprivation (partial, total, or chronic). Studies which specifically investigated the effect of substances such as caffeine or alcohol were excluded.

### Review Process

First, duplicate articles from different databases were removed. Afterwards, the title, keywords and abstract for the selected articles were checked according to the eligibility criteria by three authors (AFC, FF and GSU). In case of disagreement in the screening process, the article was re-examined to reach a consensus including the other authors (BSBG and FCS). Finally, full-text evaluation was performed.

### Assessment of Methodological Quality

The methodological quality of each article was assessed using a critical appraisal tool developed by Ku et al. (2014), based on the STROBE checklist for cohort, case-control, and cross-sectional studies (Vandenbroucke et al., 2007). The reviewers (FF, AFC and GSU) assessed the retrieved articles according to 12 questions and completed a table with the scores. A score of 2 was given if the question was satisfactorily answered in the article, a score 1 was given if it was partially answered, and a 0 given if it failed to do so, resulting in a maximum score of 24. If a paper had a score lower than 12, the paper was removed from the search.

### Summary Results

The sleep and balance control procedures and parameters were registered and tabulated to compare the experimental procedures, the data processing, and the parameters obtained.

## Results

### Search Results

**Figure 1:**
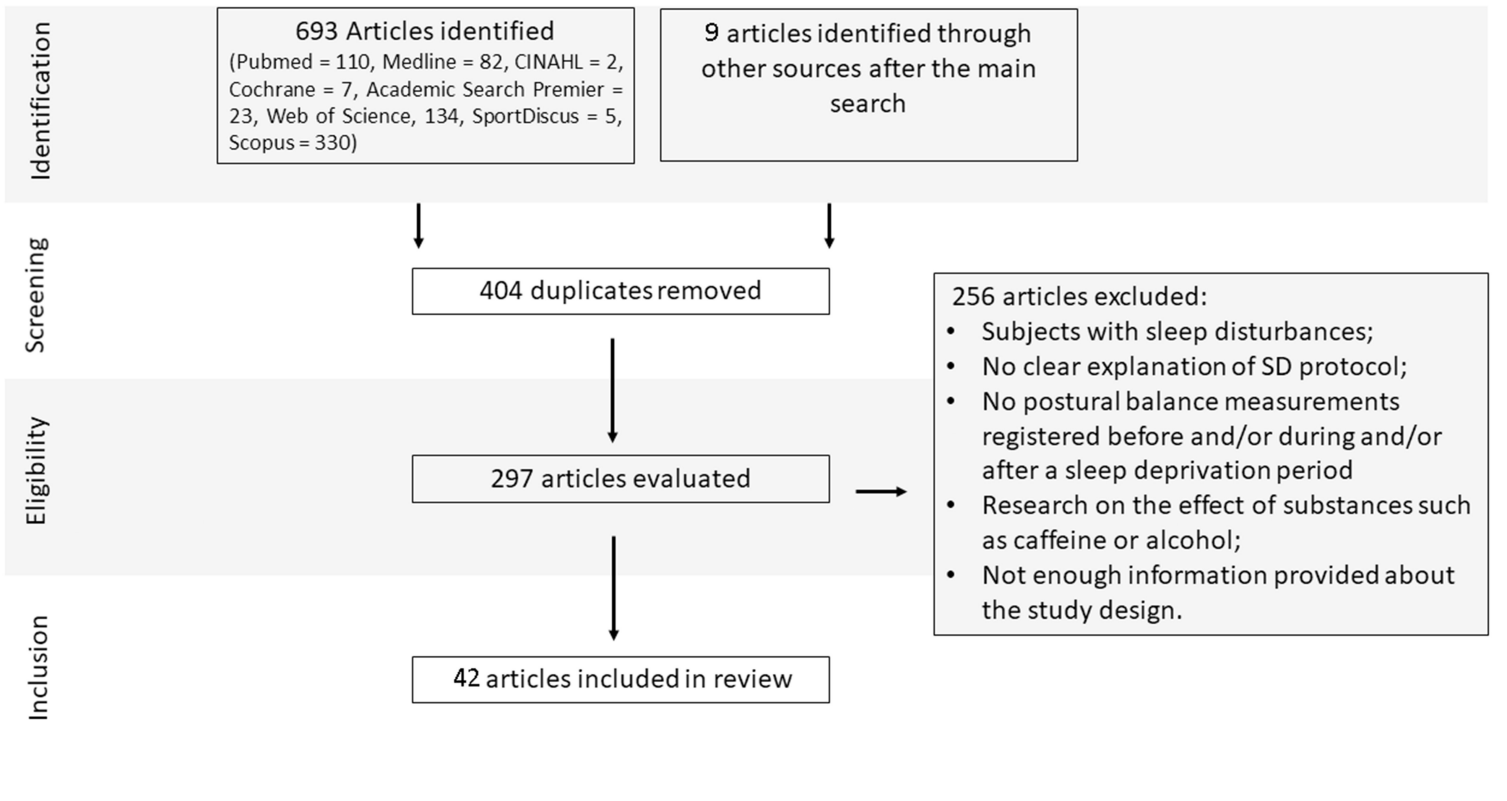
Study flow diagram and results of the literature search.

Initially, the electronic database screening search process yielded 693 articles (Pubmed = 110, Medline = 82, CINAHL = 2, Cochrane = 7, Academic Search Premier = 23, Web of Science, 134, SportDiscus = 5, Scopus = 330). The search in the remaining databases yielded several repeated articles. In addition, we identified 9 relevant papers from other sources. The screening process removed 404 duplicated articles. A total of 297 paper abstracts were evaluated to determine inclusion, and 256 papers were excluded, resulting in 42 papers included in the review (see Figure 1).

### Methodological Quality

The papers reviewed presented the goals adequately (question 1). However, the study design (question 2) had minor problems in 31 papers. The most frequent problem was the lack of assessment of the circadian rhythm of the subjects or prior sleeping conditions to set the hour of the experiments. We considered this a minor problem because the influence of these factors has only been identified in more recent studies. The studies reviewed provided complete information about practical trial (question 4), equipment design and set up (question 6), task description (question 7), adequate statistical methods (question 8), probability value (question 9), main outcomes (question 10), and conclusion (question 12). Only a few studies raised their own limitations (question 11). In summary, the quality assessment scores ranged from 15 to 24 and all the papers were thus retained for the review (Supplementary Table 1).

### Participants

In most of the studies (25 articles), the sample population is composed of individuals of both genders. Twelve articles had only male participants and five articles did not specify the sex of the participants. The physical characteristics of the participants and inclusion criteria in the reviewed articles are shown in Supplementary Table 2. Nineteen articles did not provide complete data about the participants in the study (Schlesinger et al., 1998; Nakano et al., 2001; Haeggstrom et al., 2004, 2006; Avni et al., 2006; Fabbri et al., 2006; Karita et al., 2006; Forsman et al., 2007a,b, 2008a,b, 2010a,b; Morad et al., 2007; Robillard et al., 2011a,b; Albuquerque et al., 2012; Aguiar and Barela, 2014; Cuthbertson et al., 2015; Siu et al., 2015). Most papers provided clear exclusion criteria (neurological, vestibular, musculoskeletal, psychiatric, balance, and sleep-related disorders). The number of participants varied (see Supplementary Table 2); twenty-three papers tested less than 20 participants, fourteen other papers measured between 20 and 30, while five papers had larger numbers, ranging from 55 to 63 individuals (Fabbri et al., 2006; Karita et al., 2006; Forsman et al., 2008b; Aguiar and Barela, 2014; Cheng et al., 2018). The majority of the studies assessed young adults (aged 19–29), fourteen articles included middle-aged adults (aged 30–59), and only two articles included adults over 60 years (Morad et al., 2007; Robillard et al., 2011b). The physical activity of the participants was assessed in four papers (Sobeih et al., 2006; Sargent et al., 2012; Furtado et al., 2016; Umemura et al., 2018).

Only five studies presented explicit statistical power calculation and estimation of the required sample size. The articles that show the effects of sleep deprivation on postural control, they present large effect sizes. The weighted average of main findings from these five papers resulted in an eta-squared of 0.35 which can be considered as large effect (Gribble and Hertel, 2004; Bougard et al., 2011; Albuquerque et al., 2012; Aguiar and Barela, 2015; Umemura et al., 2018).

### Sleep Deprivation Assessment

In most studies (37 out of 42), the participants had acute sleep deprivation (Supplementary Table 3). Before the balance control tests, the participants were awake between 8 and 36 h, while in two studies this period was prolonged to up to 48 h (Gribble and Hertel, 2004; Sobeih et al., 2006). Only five studies analyzed the effects of chronic SD (Karita et al., 2006; Sargent et al., 2012; Siu et al., 2015; Furtado et al., 2016; Umemura et al., 2018). A few studies checked the sleep before the tests with a self-administered questionnaire (Karita et al., 2006; Cuthbertson et al., 2015), polysomnography and sleep diary (Robillard et al., 2011a,b), only sleep diary (Aguiar and Barela, 2014; Pham et al., 2014; Batuk et al., 2020), actimetry, sleep diary and polysomnography (Sargent et al., 2012), only actimetry (Siu et al., 2015) or actimetry and questionnaires (Smith et al., 2012; Furtado et al., 2016; Umemura et al., 2018) (Supplementary Table 3). Some studies measured chronic SD using specific protocols. Sargent et al. (2012) used a forced desynchronization protocol, which is used to quantify circadian performance lengthening the day duration (28h), with 23.3h of time awake and 4.7h of sleep. Karita et al. (2006), used the self-reported total sleep time and work time to identify the subjects that suffered from chronic SD due the overtime routine. In this way, the subjects were classified in two groups prior to PC tests.

### Balance Control Assessment Under Sleep Deprivation

There were several experimental protocols that used different parameters to assess balance under SD (see Supplementary Tables 4, 5). Most of the papers measured the Center of Pressure (COP); however, there is no general agreement about which COP parameters should be used for assessing balance and SD.

Many studies used only one (Liu et al., 2001; Nakano et al., 2001; Haeggstrom et al., 2004, 2006; Avni et al., 2006; Fabbri et al., 2006; Karita et al., 2006; Forsman et al., 2007a,b, 2008a,b, 2010a,b; Bougard et al., 2011; Albuquerque et al., 2012), or two trials (Sobeih et al., 2006; Gomez et al., 2008; Patel et al., 2008; Bougard et al., 2011; Pham et al., 2014; Aguiar and Barela, 2015) per condition in each testing session (Supplementary Table 4).

Balance was most frequently assessed with a three-dimensional force platform (see Supplementary Table 4). Other studies used four vertical force plates (Avni et al., 2006), force transducers (Schlesinger et al., 1998), neuromotor test system (CATSYS 2000, Danish Product Development Ltd., Helsinger, Denmark) (Karita et al., 2006), platform-based examination systems, such as Active Balancer (EAB-100, Sakai Medical Co., Tokyo, Japan) (Ma et al., 2009) or Biodex Balance System (Biodex Systems) [18], Wii Fit Balance Board® (Nintendo, Kyoto, Japan) (Mori et al., 2017; Tietäväinen et al., 2018; Umemura et al., 2019), video-based motion measurement system with markers (Optotrak®, NDI, Ontario, Canada) (Smith et al., 2012; Aguiar and Barela, 2014) and pressure mapping under the feet (MatScan®, Tekscan Inc., Boston, USA) (Siu et al., 2015).

All the experiments included static balance control measurements. Some of them also included dynamic balance control assessment as shown in Supplementary Table 4. The COP was the most used measurement to assess balance, and it is possible to obtain global or structural parameters from it (Duarte and Freitas, 2010). The global parameters are related to the COP size in the time and frequency domains, e.g. mean sway amplitude (MSA) and root mean square (RMS) of COP excursions in the AP or ML directions (Baldan et al., 2014). The structural parameters relate epochs of the COP trajectory to motor control processes (Duarte and Freitas, 2010) and have been used to detect time awake based on a balance model (Forsman et al., 2007b). A few studies used other balance measurements, such as kinematic analysis reporting larger and faster body sway found in SD subjects with and without visual manipulation (Gomez et al., 2008; Smith et al., 2012; Aguiar and Barela, 2014).

Sensory perturbation was included in many studies, suppressing or distorting visual and/or somatosensory inputs (see Supplementary Table 5): eyes open (EO) (Gribble and Hertel, 2004; Haeggstrom et al., 2004, 2006; Sekine and Takahashi, 2005; Forsman et al., 2007a,b, 2008a,b, 2010a,b; Aguiar and Barela, 2014, 2015; Batuk et al., 2020) or eyes open and closed (EC) (Uimonen et al., 1994; Liu et al., 2001; Nakano et al., 2001; Fabbri et al., 2006; Karita et al., 2006; Sobeih et al., 2006; Morad et al., 2007; Gomez et al., 2008; Patel et al., 2008; Ma et al., 2009; Bougard et al., 2011; Robillard et al., 2011a,b; Albuquerque et al., 2012; Sargent et al., 2012; Smith et al., 2012; Pham et al., 2014; Furtado et al., 2016; Narciso et al., 2016); with external sensory stimuli (Uimonen et al., 1994; Schlesinger et al., 1998; Sekine and Takahashi, 2005; Avni et al., 2006; Gomez et al., 2008; Patel et al., 2008; Cuthbertson et al., 2015). In addition, in some studies the participants performed a dual task (Schlesinger et al., 1998; Sekine and Takahashi, 2005; Sobeih et al., 2006; Ma et al., 2009; Robillard et al., 2011a,b).

### Sleep-Related Variables

Some studies (*n* = 24), along with balance assessment, examined alertness (alpha attenuation test, critical flicker fusion frequency), sleepiness/fatigue (Stanford, Epworth and Karolinska Sleepiness Scale, or visual-analog sleepiness scale), mood (Psychomotor Global Vigor and Affect Scale) and psychomotor test (Psychomotor Vigilance Test) in addition to the sleep deprivation protocol. Two studies measured rectal temperature (Nakano et al., 2001; Sargent et al., 2012); one assessed sleep quality subjectively (Pittsburgh Sleep Quality Index) (Aguiar and Barela, 2015). Two papers applied questionnaires: sleepiness, subjective sleep quality and chronotype in addition to actimetry (Furtado et al., 2016; Umemura et al., 2018). Seventeen studies did not consider any additional variables (Supplementary Table 6).

### Sleep Deprivation and Circadian Rhythm Influence in Balance Control

**Figure 2:**
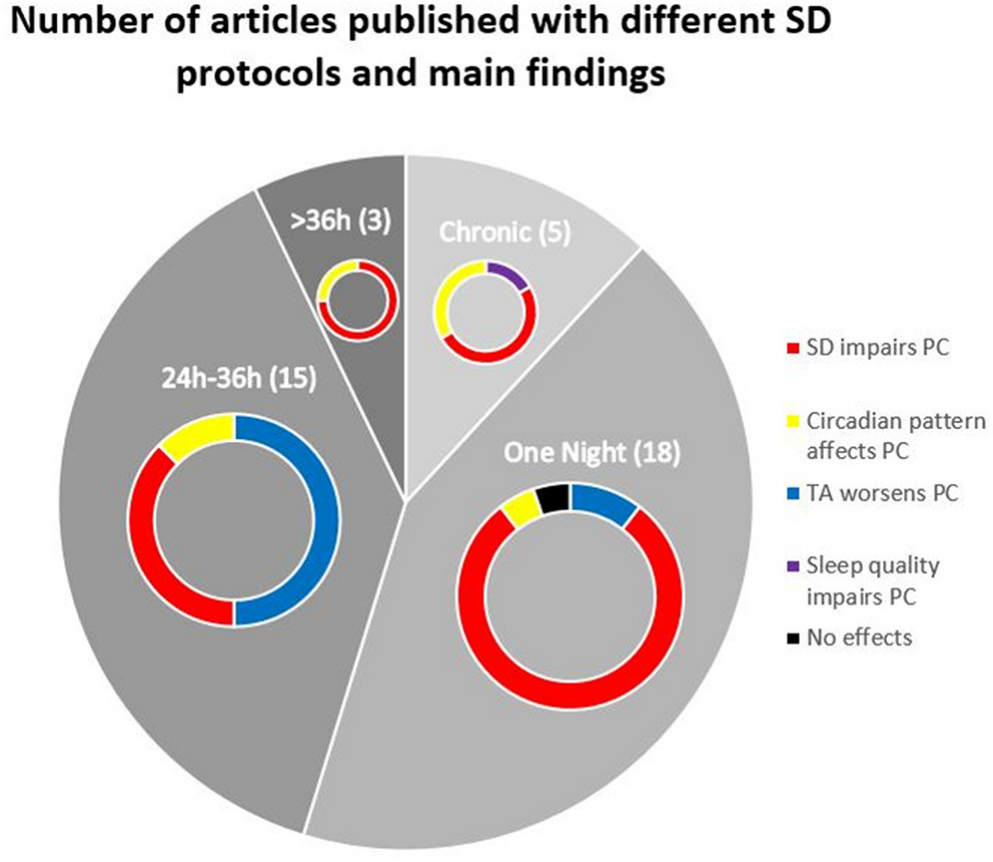
Pie chart indicating the number of papers that used different sleep deprivation protocols to assess postural control along with their main results.

The reviewed literature shows evidence that sleep deprivation, either chronic or acute, negatively affects balance control (see Figure 2). There is a consistent increase in COP area, excursion, amplitude, and variability in the antero-posterior and medio-lateral axes, after one-night of SD and similar effects were reported for chronic SD.

All the studies, except one (Uimonen et al., 1994), reported differences related to SD in a variety of static balance parameters as presented in Supplementary Table 5. Moreover, the performance for both static and dynamic tests were similarly affected by acute SD (Schlesinger et al., 1998; Avni et al., 2006; Fabbri et al., 2006; Cuthbertson et al., 2015). For instance, it was reported the deleterious effect of SD on balance control in a moving room (Aguiar and Barela, 2014, 2015). Two studies reported lower dynamic balance performance under chronic SD. There were higher COP oscillations in medio lateral, antero-posterior directions and overall (Furtado et al., 2016). Moreover, in tasks with higher cognitive demand there were higher overall COP oscillations (Umemura et al., 2018).

Several experiments removed visual (EC) or perturbed proprioceptive (foam) sensory information. The SD showed a stronger negative effect on postural control when comparing the vision feedback (EO vs. EC) than when comparing proprioceptive feedback distortion (Hard vs. Soft surface) or dual tasks.

One study reported no combined effects of SD and vision (Patel et al., 2008), while other works reported differences in balance control under SD both with and without vision (Karita et al., 2006; Gomez et al., 2008; Aguiar and Barela, 2014; Cheng et al., 2018) or only with vision (EO) (Siu et al., 2015).

Most of the studies reported differences in balance with SD (acute or chronic) and without vision (EC) (Ma et al., 2009; Robillard et al., 2011b; Sargent et al., 2012; Pham et al., 2014; Furtado et al., 2016; Umemura et al., 2019). The anterior-posterior COP excursions with EC showed significant differences due to SD (Robillard et al., 2011a; Aguiar and Barela, 2014, 2015; Siu et al., 2015; Narciso et al., 2016; Umemura et al., 2019), while the medio-lateral ones did not show significant differences in any vision condition (EO vs. EC) (Ma et al., 2009; Robillard et al., 2011b; Smith et al., 2012; Umemura et al., 2018). These results support the idea that removing the visual information is very challenging and increases the effect of SD on the sway parameters. The inclusion of foam only yielded differences in combination with EC in one study (Siu et al., 2015).

Some authors reported that the balance performance has a circadian pattern with lower values in the early morning (between 7–9 am) (Nakano et al., 2001; Avni et al., 2006) but there are large inter-subject differences (Avni et al., 2006; Morad et al., 2007; Sargent et al., 2012; Forsman et al., 2013; Furtado et al., 2016; Zouabi et al., 2016). Balance performance decreased after a threshold of 17 h of sustained wakefulness (Forsman et al., 2008b). These authors estimated a balance control performance decrease of 2.66% per hour between the 2^nd^ and 36^th^ h of wakefulness (Forsman et al., 2010b). An experimental study subjected volunteers to a simulated shift work protocol of 28h days and shift workdays starting 4h later every day. The circadian phase influenced significantly balance with eyes closed. Moreover, balance was poorer during biological nighttime (Sargent et al., 2012).

There is a group of studies that, instead of measuring the effect of SD on balance, used balance to estimate the time awake of the subject (see Supplementary Tables 4, 5) (Haeggstrom et al., 2004, 2006; Forsman et al., 2007a,b, 2008a,b, 2010a,b). They reported a 69% positive predictive value, 56% sensitivity, and 96% specificity with prediction error decaying when the time awake increased (Forsman et al., 2007b). These authors proposed a protocol to estimate if a subject was awake for more than 21 h based on global and structural parameters of COP (Pham et al., 2014).

There are some results showing that chronic SD deteriorates balance control in a similar way as acute SD (Karita et al., 2006; Sargent et al., 2012; Siu et al., 2015; Furtado et al., 2016; Forner-Cordero et al., 2018). The reduced number of studies is due to the fact that it is more difficult to monitor sleep longitudinally, in addition to being a more recent concern. On this way, there is less experimental evidence about this problem. Also, unlike the studies with acute deprivation, which have reasonably well-defined sleep deprivation protocols, research with chronic SD is usually based on the study of individuals with non-usual routines (Karita et al., 2006; Siu et al., 2015). This results in different protocols to define whether the subject is under chronic SD, which is usually based on sleep duration.

## Discussion

This review summarized the state of the art about the effects of sleep deprivation on balance control, a graphical summary can be seen in Figure 2. After a night of sleep deprivation there is a decreased performance in the control of balance, measured via the COP, especially with eyes closed. While acute SD is frequent among shift workers and clinical personnel, large part of the population suffers from chronic SD. In this respect, recent studies reported also a balance performance decrease in healthy volunteers with chronic SD (Furtado et al., 2016; Umemura et al., 2018). Moreover, some studies have used balance control parameters to estimate the time awake of the participants (Forsman et al., 2007b; Pham et al., 2014). The evidence provided shows that the sleep-wake state of the participants should be considered prior to evaluating balance control to control potential confounding factors.

### Sleep Deprivation and Sensory Perturbations

Balance control relies on visual, proprioceptive and vestibular information (Gaerlan, 2010). While vision is the most important (Uchiyama and Demura, 2009), the removal of any of them compromises balance. In this respect, SD affects the metabolism of the thalamus, cerebellum and basal ganglia, affecting sensory integration and motor coordination, and, therefore balance (Takakusaki, 2017). This would explain the more marked balance performance decrease under SD when eyes are closed (Liu et al., 2001; Nakano et al., 2001; Ma et al., 2009; Robillard et al., 2011b; Sargent et al., 2012; Pham et al., 2014; Furtado et al., 2016; Narciso et al., 2016). One of these studies reported that the closed eyes condition was more challenging for the elderly than for young adults (Robillard et al., 2011b). In this work, the authors claim that sleep disorders, common in senescence, can increase the risk of falling. They suggest that an improvement in sleep quality would help in the prevention of falls.

The inclusion of more challenging balance conditions, such as multiple sensory perturbations or complex tasks, in the experiments, may not yield differences due to two reasons. First, the increased task difficulty may increase the level of alertness, compensating temporarily the deleterious effects of SD. Second, the balance deterioration without SD induced by the experimental conditions is already large enough to mask the effect of SD (Furtado et al., 2016).

### Circadian Rhythm and Chronotype

The study of sleep is connected to the circadian oscillation; nevertheless, the assessment of circadian rhythm and chronotype were considered in a few papers. Some of them argue that circadian oscillation was respected by performing tests at similar times (Ma et al., 2009; Aguiar and Barela, 2014; Umemura et al., 2018). However, the circadian timing system has individual adjustments that vary for each subject (Frey et al., 2004; Nag and Pradhan, 2012), following the chronotype characteristics (McGowan et al., 2020). As the circadian rhythm influences balance, it is important to control this parameter to compare balance performance. The worst performance in posture control was described to occur during the Biological nighttime (Sargent et al., 2012).

A recent review analyzed the effects of chronotype and circadian rhythm on sports performance (Vitale and Weydahl, 2017). They found that morning chronotype participants had better performance in the morning tests than the evening chronotype participants. Moreover, they performed better in the afternoon tests, but not necessarily better than the morning chronotype. Therefore, it can be hypothesized that the same patterns could be found in the posture control tasks.

One important question is to know after how many hours of sleep deprivation the control of balance starts to be affected, and the answer might be linked to the mechanisms causing this performance decrease. Note that the cumulative effects of sleep deprivation cannot be studied without considering the circadian rhythm effect (Martin et al., 2018). In the study by Nakano et al. (2001), the pattern of alertness along the day was similar to the pattern of balance control performance. Therefore, the performance impairments in the morning after one night of SD can be caused by the increase of sleep pressure, resulting in lack of attention. The decrease of alertness and increase of sleepiness are more prominent behavioral consequences of the lack of sleep and affect all motor tasks, such as balance.

### Future Lines of Research

The thalamus, responsible for sensory integration, basal ganglia and cerebellum, responsible for motor coordination, show a decreased activity in glucose metabolic rate and hemodynamic response with SD (Wu et al., 1992; Drummond et al., 1999). However, more neurophysiological and neuroimaging data relating SD and balance are needed.

Only two studies addressed the effects of chronic SD on dynamic balance, underscoring the need for more research in this area. However, it seems that the consequences of chronic or acute sleep deprivation on balance control are similar, it is yet unclear if there is a dose-effect relation determined by the amount of sleep debt and if it is possible to compensate sleep loss and measure its effects on balance, opening a future line of research.

Some studies employed dual-task paradigms to assess balance under SD (Woollacott and Shumway-Cook, 2002; Gauchard et al., 2003; Fraizer et al., 2008; Allali et al., 2014). Dual-task paradigms, when used properly, can be used to simulate real-life situations, such as faced while manipulating an object or talk while standing (Forner-Cordero et al., 2007).

In addition, studies in clinical settings are needed to examine the role of sleep quality on rehabilitation and balance therapy and whether the beneficial effects of sleep translate to clinical outcomes in rehabilitative care in post stroke or spinal cord injury individuals (Gudberg and Johansen-Berg, 2015; Albu et al., 2019).

### Recommendations for Balance Control Assessment

The evidence shows that SD, either chronic or acute, affects balance control. Therefore, it is recommendable to monitor sleep and circadian rhythm for a minimum of 9 days, comprising a complete week plus 2 weekends, before the balance control experiments in order to observe the routine changes caused by social obligations throughout the week (Furtado et al., 2016; Umemura et al., 2018). With the information about the circadian rhythm and chronotype it is possible to perform the balance tests at the same phase of the circadian rhythm for all the subjects and reduce variability (Reilly, 1990; Frey et al., 2004; Gonçalves et al., 2014; Hudson et al., 2020). In addition, rehabilitation could be more effective if applied considering the circadian rhythm of the patient (Albu et al., 2019).

Although studies show gender differences in circadian expression and cognition between genders (Santhi et al., 2016), these differences are not apparent when assessing postural control in adults (Hageman et al., 1995). When comparing elderly people, in which postural control is compromised (Shaffer and Harrison, 2007), it is possible to detect gender differences, but inconclusive (Hageman et al., 1995; Sullivan et al., 2009; Wiśniowska-Szurlej et al., 2019). The lack of consensus on the results may be due to different levels of physical activity (Gauchard et al., 2003; Prioli et al., 2005). On this way, to minimize possible co-factors that may compromise the observations of the results, it is interesting to consider the assessment of physical activity levels and to have a population group with a homogeneous age, as proposed in only two studies (Furtado et al., 2016; Umemura et al., 2018).

Sleep and sleepiness can be evaluated with sleep diaries, questionnaires (Epworth Sleep Scale and Pittsburgh Sleep Quality Index) or with polysomnography. As the latter requires a heavy instrumentation of the subject (EEG, EMG, EOG), it cannot be used for several consecutive nights. Actimetry, that can be measured with a wrist watch, measures the circadian rhythm and can be correlated with questionnaires (Forner-Cordero et al., 2018). In this way, it is possible to identify the daily sleep routine as well as the daily fragmentation of rhythm, sleep quality, activity levels (Gonçalves et al., 2014; Umemura et al., 2018; Albu et al., 2019).

With respect to balance assessment, it can be recommended to measure the COP during stance and analyze its global parameters. When considering sensory perturbations, the vision conditions (EO vs. EC) yielded more differences than proprioceptive (foam) or dynamic balance tasks. Finally, the inclusion of dual tasks should be done cautiously because it may mask the deleterious effects of SD or increase the alertness and attention levels of the participants.

## Data Availability Statement

The original contributions presented in the study are included in the article/Supplementary Material, further inquiries can be directed to the corresponding author/s.

## Author Contributions

AF-C, FF, and GU drafted the manuscript. BG and FS contributed to the selection criteria. FF and AF-C developed the search strategy, paper quality assessment, and data extraction. GU and FS updated the search and data extraction. BG and GU revised the sleep assessment techniques. All authors have made substantial contribution to the manuscript. All authors read, provided feedback, and approved the final version.

## Funding

This work was receive funding from the Office of Naval Research Global (ONR-G Grant N62909-13-1-N278) is acknowledged. AF-C thanks the Brazilian National Council of Scientific and Technological Development grant (CNPq 311055/2016-8).

## Conflict of Interest

The authors declare that the research was conducted in the absence of any commercial or financial relationships that could be construed as a potential conflict of interest.

## Publisher's Note

All claims expressed in this article are solely those of the authors and do not necessarily represent those of their affiliated organizations, or those of the publisher, the editors and the reviewers. Any product that may be evaluated in this article, or claim that may be made by its manufacturer, is not guaranteed or endorsed by the publisher.
