# Supplementary material for: Is Balance Control Affected by Sleep Deprivation? A Systematic Review of the Impact of Sleep on the Control of Balance
Source: Front Neurosci. 2022 May 16;16:779086. doi: 10.3389/fnins.2022.779086 (PMC9150847; doi:10.3389/fnins.2022.779086)
Supplement: Supplementary file 1 [file Table_1.DOCX]

TABLES

Supplementary Material

Table 1. Methodological quality of the articles

| **Article** | **Question 1** | **Question 2** | **Question 3** | **Question 4** | **Question 5** | **Question 6** | **Question 7** | **Question 8** | **Question 9** | **Question 10** | **Question 11** | **Question 12** | **Total** |
| --- | --- | --- | --- | --- | --- | --- | --- | --- | --- | --- | --- | --- | --- |
| Aguiar and Barela 2014 | 2 | 2 | 1 | 2 | 0 | 2 | 2 | 2 | 2 | 2 | 2 | 2 | 21 |
| Aguiar and Barela 2015 | 2 | 2 | 2 | 2 | 0 | 2 | 2 | 2 | 2 | 2 | 2 | 2 | 22 |
| Albuquerque et al. 2012 | 2 | 1 | 1 | 2 | 2 | 2 | 2 | 2 | 2 | 2 | 0 | 2 | 20 |
| Avni et al. 2006 | 2 | 2 | 1 | 2 | 0 | 2 | 2 | 2 | 2 | 2 | 0 | 2 | 19 |
| Batuk et al.2020 | 2 | 2 | 2 | 2 | 0 | 2 | 2 | 2 | 2 | 2 | 2 | 2 | 22 |
| Bougard et al. 2011 | 2 | 1 | 2 | 2 | 2 | 2 | 2 | 2 | 2 | 2 | 0 | 2 | 21 |
| Cheng et al 2018. | 2 | 2 | 2 | 2 | 2 | 2 | 2 | 2 | 2 | 2 | 2 | 2 | 24 |
| Cuthbertson et al. 2015 | 2 | 1 | 1 | 2 | 1 | 2 | 2 | 2 | 2 | 2 | 2 | 2 | 21 |
| Fabbri et al. 2006 | 2 | 1 | 1 | 2 | 2 | 2 | 2 | 2 | 2 | 2 | 2 | 2 | 22 |
| Forsman et al. 2007a | 2 | 1 | 1 | 2 | 1 | 2 | 2 | 2 | 2 | 2 | 0 | 2 | 19 |
| Forsman et al. 2007b | 2 | 1 | 1 | 2 | 1 | 2 | 2 | 2 | 2 | 2 | 0 | 2 | 19 |
| Forsman et al. 2008a | 2 | 1 | 1 | 2 | 1 | 2 | 2 | 2 | 2 | 2 | 0 | 2 | 19 |
| Forsman et al. 2008b | 2 | 2 | 1 | 2 | 1 | 2 | 2 | 2 | 2 | 2 | 2 | 2 | 22 |
| Forsman et al. 2010a | 2 | 1 | 1 | 2 | 1 | 2 | 2 | 2 | N | 2 | 2 | 2 | 19 |
| Forsman et al. 2010b | 2 | 1 | 2 | 2 | 1 | 2 | 2 | 2 | 2 | 2 | 0 | 2 | 20 |
| Furtado et al. 2016 | 2 | 2 | 2 | 2 | 1 | 2 | 2 | 2 | 2 | 2 | 2 | 2 | 23 |
| Gomez et al. 2008 | 2 | 1 | 2 | 2 | 0 | 2 | 2 | 2 | 2 | 2 | 0 | 2 | 19 |
| Gribble and Hertel 2004 | 2 | 1 | 2 | 2 | 1 | 2 | 2 | 2 | 2 | 2 | 2 | 2 | 21 |
| Haeggstrom et al. 2004 | 2 | 1 | 1 | 2 | 2 | 2 | 2 | 2 | N | 2 | 0 | 2 | 18 |
| Haeggstrom et al. 2006 | 2 | 1 | 1 | 2 | 2 | 2 | 2 | 2 | N | 2 | 0 | 2 | 18 |
| Karita et al. 2006 | 1 | 1 | 1 | 2 | 1 | 2 | 2 | 2 | 1 | 1 | 2 | 2 | 18 |
| Liu et al. 2001 | 2 | 1 | 2 | 2 | 2 | 2 | 2 | 2 | 2 | 2 | 0 | 2 | 21 |
| Ma et al. 2009 | 2 | 1 | 2 | 2 | 1 | 2 | 2 | 2 | 2 | 2 | 1 | 2 | 21 |
| Mantua et al . 2020 | 2 | 1 | 1 | 0 | 2 | 2 | 2 | 2 | 2 | 2 | 2 | 2 | 20 |
| Martin et al. 2018 | 2 | 2 | 1 | 1 | 1 | 2 | 2 | 2 | 2 | 2 | 1 | 2 | 20 |
| Morad et al. 2007 | 2 | 1 | 1 | 2 | 0 | 1 | 2 | 2 | 2 | 2 | 0 | 2 | 17 |
| Mori et al. 2017 | 2 | 1 | 1 | 1 | 2 | 1 | 2 | 2 | 2 | 2 | 1 | 2 | 19 |
| Nakano et al. 2001 | 1 | 1 | 1 | 2 | 0 | 1 | 2 | 1 | 2 | 2 | 0 | 2 | 15 |
| Narciso et al. 2016 | 2 | 2 | 2 | 2 | 1 | 2 | 2 | 2 | 2 | 2 | 1 | 2 | 22 |
| Patel et al. 2008 | 2 | 1 | 2 | 2 | 0 | 2 | 2 | 2 | 2 | 2 | 0 | 2 | 19 |
| Pham et al. 2014 | 2 | 2 | 2 | 2 | 0 | 2 | 2 | 2 | 2 | 2 | 2 | 2 | 22 |
| Robillard et al. 2011a | 2 | 1 | 1 | 2 | 2 | 2 | 2 | 2 | 2 | 2 | 2 | 2 | 22 |
| Robillard et al. 2011b | 2 | 1 | 1 | 2 | 2 | 2 | 2 | 2 | 2 | 2 | 2 | 2 | 22 |
| Sargent et al. 2012 | 2 | 2 | 2 | 2 | 2 | 2 | 2 | 2 | 2 | 2 | 2 | 2 | 24 |
| Schlesinger et al. 1998 | 2 | 1 | 1 | 2 | 0 | 1 | 2 | 2 | 2 | 2 | 2 | 2 | 19 |
| Sekine and Takahashi 2005 | 2 | 1 | 1 | 2 | 2 | 1 | 1 | 2 | 2 | 2 | 1 | 2 | 19 |
| Siu et al. 2015 | 2 | 1 | 1 | 1 | 0 | 2 | 2 | 2 | 2 | 2 | 1 | 2 | 18 |
| Smith et al. 2012 | 2 | 1 | 2 | 2 | 2 | 2 | 2 | 2 | 2 | 2 | 0 | 2 | 21 |
| Sobeih et al. 2006 | 2 | 1 | 2 | 2 | 0 | 2 | 2 | 2 | 2 | 2 | 0 | 2 | 19 |
| Tietäväinen et al. 2013 | 2 | 1 | 2 | 2 | 0 | 2 | 2 | 2 | 2 | 2 | 0 | 2 | 19 |
| Uimonen et al. 1994 | 2 | 1 | 1 | 2 | 2 | 2 | 2 | 2 | 2 | 2 | 0 | 2 | 20 |
| Umemura et al. 2018 | 2 | 1 | 2 | 2 | 1 | 2 | 2 | 2 | 2 | 2 | 1 | 2 | 21 |
| Umemura et al. 2019 | 2 | 1 | 1 | 1 | 1 | 2 | 1 | 2 | 2 | 2 | 1 | 2 | 18 |
|  |  |  |  |  |  |  |  |  |  |  |  |  |  |

2:Yes; 1 = Limited detail; 0 = no, N = Not applicable.

**Table 2.** Characteristics of the participants

| Study | N of participants | Age (years) | Inclusion criteria |
| --- | --- | --- | --- |
| Aguiar and Barela 2014 | SDG: 30; CG: 30 | SDG: 24.93; CG: 27.16 | Normal or corrected-to-normal vision, no musculoskeletal impairment that could interfere in the postural task, and no diagnosed sleep disorders |
| Aguiar and Barela 2015 | SDG: 15; CG: 15 | SDG: 23.60; CG: 27.35 | No diagnosed sleep disorders; no musculoskeletal impairments that could interfere in the postural control task; and normal or corrected-to-normal vision |
| Albuquerque et al. 2012 | 17 | 36.7 | No sleep-related diseases or disorders of circadian sleep-wake rhythm |
| Avni et al. 2006 | 10 | 16-33 (range) | No postural problems, minor orthopedic abnormalities, and suspected sleep disorders |
| Bougard et al. 2011 | 20 | 24.6 | Indifferent chronotype (HO questionnaire) |
| Batuk et al.2020 | 31 | 24.96 | No postural problems or visual disorders |
| Cheng et al 2018 | 66 | Cohort1= 23.3;  Cohort2= 21.4 | No bone fractures, muscle injuries, vestibular dysfunction, or  other diseases affecting upright posture or balance equilibrium in the 3  months preceding our study. |
| Cuthbertson et al. 2015 | 10 | 29 | No migraines, recent ear infection, visual impairment, peripheral neuropathy, pregnancy, musculoskeletal injuries, vestibular disorders or neurologic problems; |
| Fabbri et al. 2006 | 55 | F= 23.28/ M=23.87 | _____ |
| Forsman et al. 2007a | 21 | 20-37 (range) | _____ |
| Forsman et al. 2007b | 20 | 20-37 (range) | _____ |
| Forsman et al. 2008a | 12 | 21-38 (range) | _____ |
| Forsman et al. 2008b | 63 (33 in experiment I) | 20-38 (range) | No smoking, diagnosed balance- or sleep disorders, current leg- or back injuries, or current medication affecting sleepiness |
| Forsman et al. 2010a | 20 | 20-37 (range) | No smoking, alcohol abuse, regular medication, and diagnosed balance, or sleep disorders |
| Forsman et al. 2010b | 12 | 21-38 (range) | _____ |
| Furtado et al. 2016 | 30 | 18-29 (range) | No history of musculoskeletal, rheumatological, neurological, visual, vestibular, psychiatric diseases or diabetes mellitus. 18.5 < BMI ≤ 30. |
| Gomez et al. 2008 | 18 | 23.8 | No history of balance problems, central nervous disease or injury to the musculoskeletal system |
| Gribble and Hertel 2004 | 24 | F=20/M=21 | No vestibular disorders, head or lower extremity injury in previous 12 months |
| Haeggstrom et al. 2004 | 20 | 20-37 (range) | No diagnosed balance disorder |
| Haeggstrom et al. 2006 | 21 | 20-37 (range) | Non-smokers; balance disorder diagnosis |
| Karita et al. 2006 | 60 | Longer OW= 29; Shorter OW=28.7; CG= 32.1 | No neurologic, otolaryngologic, orthopedic and vestibular disorders, or medical history of articular rheumatism, arthralgia or diabetes mellitus |
| Liu et al. 2001 | 7 | 23.5 | No musculoskeletal or neurological impairments; neither extreme morning nor evening type |
| Ma et al. 2009 | 16 | 20.75 | No self-reported mental, nervous or sleep disorders |
| Martin et al. 2018 | 19 | 20-25 (range) | No history of night or shift work, no transmeridian travels before the study, no reported sleep complaints, sleep disorder, vestibular condition, hearing loss or proprioceptive deficits. |
| Morad et al. 2007 | 12 | 20-60 (range) | No postural problems, minor orthopedic abnormalities, and suspected sleep disorders |
| Mori et al. 2017 | 7 | 21-24 | Healthy individuals |
| Nakano et al. 2001 | 8 | 23.8 | _____ |
| Narciso et al. 2016 | 20 | 25-55 (range) | No sleep disorders, visual and hearing impairments, neurological/degenerative brain diseases, musculoskeletal injuries; alcohol consumption 24h prior to the measurements; if the participant worked daytime shifts and double shifts; or if he/she slept during the shift |
| Patel et al. 2008 | 18 | 23.8 | No previous history of central nervous diseases |
| Pham et al. 2014 | 14 | 23 | No self-reported sleep disorders or postural deficits |
| Robillard et al. 2011a | 13 | 25 | No history of visual, sleep, auditory, postural, vestibular, psychiatric, and/or neurological impairment; BMI>30 |
| Robillard et al. 2011b | 15 young;  15 older adults | Young=24; older=64 | No history of visual, sleep, auditory, postural, vestibular, psychiatric, and/or neurological impairment; BMI >30 |
| Sargent et al. 2012 | 14 | 21.38 | No neurological, psychiatric, endocrine, and sleep disorders. No high intake of caffeine or alcohol. Non-smokers, medication free. No shift work or transmeridian travel in the last 3 months |
| Schlesinger et al. 1998 | 5 | 20 | No history of sleep vestibular, or neurological disorders |
| Sekine and Takahashi 2005 | 10 | 21.4 | No abnormal hearing and vestibular function and history of trauma |
| Siu et al. 2015 | 5 | 54 | The participant medical screening, having no health conditions that could affect their participation in the study. |
| Smith et al. 2012 | 9 | 21.56 | No sick, drug users that could impede balance abilities. No transmeridian travel or shiftwork 1 week prior to the study. No excessive caffeine consumption, or self-related sleep difficulties |
| Sobeih et al. 2006 | 16 | 31.38 | No conditions affecting balance: dizziness, tremor, alcoholism, neurologic or vestibular disorders, diabetes or chronic back pain |
| Tietäväinen et al. 2013 | 15 | 22-33 (range) | No sleep or balance disorders, medical conditions or medication, or injuries. |
| Umemura et al. 2018 | 30 | 18-29 (range) | No history of musculoskeletal, rheumatological, neurological, visual, vestibular, psychiatric diseases or diabetes mellitus. 18.5 < BMI ≤ 30. |
| Umemura et al. 2019 | 8 | - | No psychiatric or sleep disorders during the previous year and  consumption of drugs that may affect sleep |
| Uimonen et al. 1994 | 23 | 30 (median) | No history of vertigo, central nervous disorders and ear disease. |

F: female; M: male; BMI: body mass index; SDG: Sleep Deprivation Group; CG: Control Group; OW: Overtime Work; _____:Not specified

**Table 3.** Experimental sleep conditions and assessment parameters

|  | Sleep conditions  (factors) | | Sleep parameters assessment | |
| --- | --- | --- | --- | --- |
| Study | Sleep deprivation | Hours without sleep | Objective | Subjective |
| Aguiar and Barela 2014 | Acute | 27h (20:00-11:00) | _____ | Sleep diary (only in the night before the sleep deprivation period) |
| Aguiar and Barela 2015 | Acute | 27h (20:00-11:00 | _____ | Sleep diary (3 days before the sleep deprivation period) |
| Albuquerque et al. 2012 | Acute | (1 night) | _____ | _____ |
| Batuk et al.2020 | Acute | 24h | _____ | Sleep diary (3 days before the tests) |
| Avni et al. 2006 | Acute | 25h (21:00-10:00) | _____ | _____ |
| Bougard et al. 2011 | Acute | (1 night) | _____ | _____ |
| Cheng et al 2018 | Acute | 40h (06:00-22:00) | _____ | Pittsburgh Sleep Quality Index  Stanford Sleepiness Scale |
| Cuthbertson et al. 2015 | Acute | (1 night) | _____ | Questionnaire of sleep habits (before tests) |
| Fabbri et al. 2006 | Acute | (1 night) | _____ | _____ |
| Forsman et al. 2007a | Acute | > 32h (1 night) | _____ | _____ |
| Forsman et al. 2007b | Acute | 28h (8:00-12:00) | _____ | _____ |
| Forsman et al. 2008a | Acute | 36h (8:00-18:00) | _____ | _____ |
| Forsman et al. 2008b | Acute | 28h (1 night) | _____ | _____ |
| Forsman et al. 2010a | Acute | 28h (8:00-12:00) | _____ | _____ |
| Forsman et al. 2010b | Acute | 36h (8:00-18:00) | _____ | _____ |
| Furtado et al. 2016 | Chronic | Normal routine over 9 days | Actigraphy | Pittsburgh Sleep Quality Index  Epworth Sleepiness Scale |
| Gomez et al. 2008 | Acute | 24h or 36h | Portable EEG | _____ |
| Gribble and Hertel 2004 | Acute | 48h | _____ | _____ |
| Haeggstrom et al. 2004 | Acute | 30-36h | _____ | _____ |
| Haeggstrom et al. 2006 | Acute | 30-36h (1 night) | _____ | _____ |
| Karita et al. 2006 | Chronic | Routinely over days | _____ | Self-administered questionnaire and interview with specialist |
| Liu et al. 2001 | Acute | 1 night | _____ | _____ |
| Ma et al. 2009 | Acute | 24h (1 night) | _____ | _____ |
| Martin et al. 2018 | Acute | 1 night | Actigraphy | Karolinska Sleepiness Scale |
| Morad et al. 2007 | Acute | 26h (8:00-10:00) | _____ | _____ |
| Mori et al. 2017 | Acute | 36h | _____ | Stanford Sleepiness Scale |
| Nakano et al. 2001 | Acute | 19h (15:00-9:00) | _____ | _____ |
| Narciso et al. 2016 | Acute | 12h | Actigraphy | Sleep diary (for 5 days) and  Karolinska Sleepiness Scale |
| Patel et al. 2008 | Acute | 24h or 36h | Portable EEG | _____ |
| Pham et al. 2014 | Acute | 36h (1 night) | _____ | _____ |
| Robillard et al. 2011a | Acute | 26h (1 night) | Polysomnography | Pittsburgh Sleep Diary (1 week before tests) |
| Robillard et al. 2011b | Acute | 26h (1 night) | Polysomnography | Pittsburgh Sleep Diary (1 week before tests) |
| Sargent et al. 2012 | Chronic | Normal routine over 10 days | Actigraphy and polysonmnography | Sleep diaries |
| Schlesinger et al. 1998 | Acute | 1 night | _____ | _____ |
| Sekine and Takahashi 2005 | Acute | 1 night | _____ | _____ |
| Siu et al. 2015 | Chronic | Net sleep = sleep hours before balance - mean hours during previous week | Actigraphy | _____ |
| Smith et al. 2012 | Acute | 1 night | Actigraphy | Pittsburgh Sleep Diary (6-day period prior to testing day) |
| Sobeih et al. 2006 | Acute | Variable (12-48h) | _____ | _____ |
| Tietäväinen et al. 2013 | Acute | 24h (1 night) | _____ | _____ |
| Umemura et al. 2018 | Chronic | Normal routine over 9 days | Actigraphy | Pittsburgh Sleep Quality Index  Epworth Sleepiness Scale |
| Umemura et al. 2019 | Acute | 1 night | _____ | _____ |
| Uimonen et al. 1994 | Acute | 24h (1 night) | _____ | _____ |

_____: not specified

**Table 4.** Balance protocol data

| Study | Balance assessment | Total number of trials | Trial duration | Instrumentation | Sampling  rate (Hz) | Dual  Task |
| --- | --- | --- | --- | --- | --- | --- |
| Aguiar and Barela 2014 | Dynamic | 4 (1 trial: static room; 3 trials: room moved) | 60 s | Optotrak® cameras | 100 | _____ |
| Aguiar and Barela 2015 | Dynamic | 7 (in room with different configurations) | 60 s | Optotrak® cameras | 100 | _____ |
| Albuquerque et al. 2012 | Static | 4 (EO/EC, with/without SD) | 60 s | Force platform | 100 | _____ |
| Avni et al. 2006 | Dynamic | 12 times in a 25h. interval | 48 s | Tetrax® Interactive Balance System | 32 | _____ |
| Batuk et al.2020 | Dynamic | 3 trials in each 6 conditions before and after SD | 20s | CDP system (Neurocom) | ____ | mPT, DVA, GST |
| Bougard et al. 2011 | Static | 16 (at 6:00, 10:00, 14:00, 18:00h; EO/EC, with/without SD) | 51.2 s | Force platform | 40 | _____ |
| Cheng et al 2018. | Static | 12 (4 times in a 40h interval with 3 different conditions) | _____ | Force plate (Active Balancer EAB-100-Sakai®; Tetrax® Interactive) | _____ | _____ |
| Cuthbertson et al. 2015 | Dynamic | 43 (2 in CTSIB - before/after; CDP: 18 in MTC, 5 in ADT, 18 in SOT - 3 repetitions in 6 conditions) | CTSIB: 30 s; CDP: by equipment | Force platform (EquiTest ® - CDP). | _____ | _____ |
| Fabbri et al. 2006 | Dynamic | 4 (EO/EC, with/without SD) | 60 s | Normalized balance platform | _____ | _____ |
| Forsman et al. 2007a | Static | 15 (every 2 h in 48h interval, and 1 with the hour choose by the volunteer) | 30 s | Force platform | 1000 | _____ |
| Forsman et al. 2007b | Static | 16 (every 2h in 32h interval) | 30 s | Force platform | 1000 | _____ |
| Forsman et al. 2008a | Static | 18 (every 2h in 36h interval) | 30 s | Force platform | 1000 | _____ |
| Forsman et al. 2008b | Static | 21 (14 - every 2h in 28h interval); 7 (3 initial plus 1 every week for a month); | 30 s | Force platform | 1000 | _____ |
| Forsman et al. 2010a | Static | 29 (18- every 2 h in 36h interval; 7 - every day for a week; 4 - every week for a month) | 30 s | Force platform | 1000 | _____ |
| Forsman et al. 2010b | Static | 14 (every 2h in 28h interval) | 30 s | Force platform | _____ | _____ |
| Furtado et al. 2016 | Dynamic | 42 (DT1 - 15: 3 blocks with 5 repetitions; DT2 -15: 3 blocks with 5 levels; DT3 - 4: 2 repetitions in 2 levels; STSI - 8: 2 repetitions in 4 conditions). | 30 s | Biodex Balance System® | _____ | _____ |
| Gomez et al. 2008 | Static | 6 (two conditions: without SD, 24h of SD, 36h of SD) | 235 s | Zebris™ system | _____ | _____ |
| Gribble and Hertel 2004 | Static | 24 (3 repetitions every 6h in 48h interval) | 15 s | Force platform | 50 | _____ |
| Haeggstrom et al. 2004 | Static | 18 (every 2h in 36h interval) | 30 s | Force platform | 1000 | _____ |
| Haeggstrom et al. 2006 | Static | 18 (every 2h in 36h interval) | 30 s | Force platform | 1000 | _____ |
| Karita et al. 2006 | Static | 20 (5 repetitions with EO/EC, repeated after three days | 60 s | Neuromotor Test System - CATSYS 2000 | _____ | _____ |
| Liu et al. 2001 | Static | 16 (EO/EC: hourly from 22:00h to 04:00h and 8:30h) | 30 s | Force platform | 20 | _____ |
| Ma et al. 2009 | Static | 4 (with EO/EC, with/without SD) | 30 s | EAB-100 balance examination system | _____ | SDT |
| Martin et al. 2018 | Static | 2 (with EO/EC) | 30 s | Force plate (Synapsys® posturography) | 100 | _____ |
| Morad et al. 2007 | Static | 10 (in 26h interval) | 48 s | Tetrax® Interactive Balance System | 32 | _____ |
| Mori et al. 2017 | Static | 26 (each 1.5h with EO/EC) | 60 s | WiiFit® Balance Board | 100 | _____ |
| Nakano et al. 2001 | Static | 38(every 1h from 15:00h to 9:00h with EO/EC) | 40 s | Force platform | _____ | _____ |
| Narciso et al. 2016 | Static | 8 (2 repetitions in two tests with EO/EC) | 30 s | Force platform | 100 | _____ |
| Patel et al. 2008 | Static | 6 (tests with EO/EC: without SD, after 24h SD, after 36h SD) | 235 s | Force platform | 50 | _____ |
| Pham et al. 2014 | Static | 144 (18 trial every 2 h in 36h interval: 4 different conditions with 2 repetitions per trial) | 40 s | Force platform | 100 | _____ |
| Robillard et al. 2011a | Static | 12 (with/without SD in 6 different conditions) | 120 s | Force platform | 256 | ICAT |
| Robillard et al. 2011b | Static | 12 (with/without SD in 6 different conditions) | 120 s | Force platform | 256 | ICAT |
| Sargent et al. 2012 | Static | 146 (each 1.5h in the 13 days protocol with EO/EC) | 60 s | Force platform | _____ | _____ |
| Schlesinger et al. 1998 | Dynamic | 108 (54: 6 trials in each condition, with /without SD) | 60 s | Floor force transducers | 100 | RT, IRT |
| Sekine and Takahashi 2005 | Dynamic | 3 (morning; morning after SD; afternoon after SD). | _____ | Force platform | _____ | MAT |
| Siu et al. 2015 | Static | 18 (3 in each condition) | 10 s | Pressure mapping mat | _____ | _____ |
| Smith et al. 2012 | Static | 28 (every hour in 14h interval with EO/EC) | 180 s | Force platform | 100 | _____ |
| Sobeih et al. 2006 | Dynamic | 27(3 platform conditions; 3 Personal Protective Equipment conditions; 3 time on work shift conditions) | 30 s | Force platform | 50 | Lift 5-lb. (4 times) |
| Tietäväinen et al. 2013 | Static | 100 (every hour in 24h interval with 4 repetition each) | 30 s | WiiFit® Balance Board | 60 | _____ |
| Umemura et al. 2018 | Dynamic | 16 (2 in each of 8 conditions) | 30 s | Biodex® Balance Syst. | _____ | _____ |
| Umemura et al. 2019 | Static | 8 (2 in each of 4 conditions) | 60 s | WiiFit® Balance Board | _____ | _____ |
| Uimonen et al. 1994 | Static | 20 (with EO/EC: weekly in first month; 1 per month for 4 months; 2 repetitions after SD) | 55 s | Force platform | 33.3 | _____ |

ADT: Adaptation test; CDP: computerized dynamic posturography; CTSIB: Clinical Test of Sensory Integration and Balance; DT: Dynamic Test; DVA:Dynamic visual acuity test; EC: eyes closed, EO: eyes open; GSA: Gaze stabilization test; ICAT: Interference and control auditory tasks; IRT: inhibitor reaction task; MAT: Mental arithmetic test;mPT: Minimal visual perception time test; MTC: motor control test; RT: Reaction task; SD: sleep deprivation; SDT: Sternberg dual task; SOT: sensory organization test; STSI: Static Test–Sensory integration; _____: not specified.

**Table 5.** Balance control outcome data

| Study | COP | Kinematics | **Outcome** |
| --- | --- | --- | --- |
| Aguiar and Barela 2014 | MSA(a-p) and velocity in AP direction | Coherence, gain, phase, position and velocity | Larger and faster body sway found in sleep deprived subjects with and without visual manipulation. |
| Aguiar and Barela 2015 | MSA(a-p) and velocity in AP direction | Gain, phase, position and velocity | After the abrupt change in visual cues, larger amplitude, and higher velocity of the room, the influence of room motion on body sway decreased less in sleep deprived than control subjects. |
| Albuquerque et al. 2012 | COPV, amplitude, mean frequency and velocity of AP and ML displacements | _____ | An interaction effect of time of day and type of shift in both conditions EO and EC for amplitude of ML movements was observed. Balance was affected by a night of work. |
| Avni et al. 2006 | SRSS, FI, Sway intensity different frequency bands, | _____ | Fatigue index had circadian pattern with an instability peak in early morning. Medium low frequencies of sway were most affect by fatigue |
| Batuk et al.2020 | SOT parameters | _____ | SD reduced the performance SOT parameters, but not for tests done with eyes open with fixed platform. |
| Bougard et al. 2011 | C90, LFS ratio, RI | _____ | Balance fluctuates diurnally: at 6 am there was no change due to SD; at 10 am and 2 pm, C90 was higher following SD; at 6 pm LFS ratio was higher whereas C90 decreased to the 6 am level. |
| Cheng et al 2018. | WPL, COPA, StDev-L, StDev-AP, MSA(a-p), MSA(m-l). Sway intensity at different frequency bands | _____ | For COPA and StDev-AP, balance deteriorates after 16 hours of SD with EO in solid platform and after 28 hours for EC in solid and foam platform; for WPL and StDev-ML, the deterioration was observed after 28 hours of SD |
| Cuthbertson et al. 2015 | SOT parameters | _____ | SOT: visual dependence condition score increased significantly from pre- to postcall; performance on SOT condition 5 decreased. |
| Fabbri et al. 2006 | StDev-L, StDev-AP, SS, SKL, LFS Romberg’s index | _____ | EO: lower scores for StDev-L, StDev-AP SS, SKL. SKL at 22h was lower than at 8h. LFS with EO was higher than EC at 22h and also with EO at 8h; Romberg’s index was higher at 22h than at 8h |
| Forsman et al. 2007a | FD, TOC, and MCSA | _____ | TA was estimated from 18 s posturographic trials with 5 h accuracy. With increasing TA, even shorter trials estimated the TA with the same accuracy |
| Forsman et al. 2007b | FD, MCSA and TOC. | _____ | Training set: Regression analysis showed a linear correlation between balance and time awake (TA) 60% variance. Test set: For 1h increase of TA, predicted TA increased 1.02 h |
| Forsman et al. 2008a | CT interval, calculated from A-P COM movement | _____ | Regression analysis showed a linear correlation between balance and TA (65% of the variance) and between estimated and true TA (80% of the variance). |
| Forsman et al. 2008b | TOC | _____ | Regression analysis in the sustained waking condition revealed a linear correlation between balance and TA that accounted for 60% of the balance variance |
| Forsman et al. 2010a | FD, MCA, and FT | _____ | The new balance score was more sensitive than its components to sleepiness-related balance decrements during a 28-h period. |
| Forsman et al. 2010b | FT | _____ | Between the 2nd and 36th hour of SD balance impairment was 2.66% per hour; Linear correlation between the estimated and the actual TA (70% of the variance). |
| Furtado et al. 2016 | Stability index of COP: APSI, MLSI, OSI | _____ | Participants with lower sleep quality had significantly worse balance parameters with EO in MLSI and OSI and with EC in OSI, APSI and MLSI. |
| Gomez et al. 2008 | Normalized variance PS of the head, shoulder, hip, knee. | Linear motion of head, shoulder, hip, knee and ankle | SD reduced the ability to adapt head, shoulder, and hip motion with EO and EC. Postural performance did not deteriorate from 24 to 36 h of SD |
| Gribble and Hertel 2004 | COPA and COPV | _____ | COPV changed with smallest values at midnight on both days. In the first 24-hr period, balance worsened |
| Haeggstrom et al. 2004 | FD, MCFA, and TOC. | _____ | SD estimation accuracy was significantly reduced with the reduction of measurement time. |
| Haeggstrom et al. 2006 | 163 posturographic parameters | _____ | Training: parameters identified FD of COP, frequency and amplitude ML, critical time (CT)-interval. Test: accuracy better than 5 h was obtained for 80% of the cases |
| Karita et al. 2006 | COPA and COPV | _____ | EO and EC: COPA was larger for longer overtime workers than shorter ones and control groups; COPV was larger than control group. |
| Liu et al. 2001 | RA, RMS-AP,  MF-AP, MF-L, | _____ | RA, RMS-AP, MF-AP and MF-L increased after midnight with a peak at 4h. Increases in RA and MF-AP were larger with eyes closed |
| Ma et al. 2009 | WPL, total COP path length, COPA, RA, UAPL, StDev-L, StDev-AP, MD-L and MD-AP, RI. | _____ | Before and after a 24 awake, EC: WPL, CA, RA, StDev-AP increased/UAPL decreased. StDev-L/EO was higher after sustained wakefulness. |
| Martin et al. 2018 | COPA, total displacement | _____ | Balance decreases after sleep deprivation but remains stable throughout the day. |
| Morad et al. 2007 | Stability index and sway intensity | _____ | EC: stability and sway showed a circadian pattern with a peak at morning;  EO: changes in frequency band is related to vestibular function. |
| Mori et al. 2017 | WPL, COPA, UAPL and sparse density. | _____ | Balance worsening after 22.5 hours of SD in trials with EC |
| Nakano et al. 2001 | PS product of maximum AP and ML sway | _____ | PS with EC increased in early morning following the attention reduction. |
| Narciso et al. 2016 | MSA(a-p), MSA(m-l), TSD | _____ | MSA (a-p) and TSD with EO and TSD with EC increased significantly after12h of SD |
| Patel et al. 2008 | Normalized variance of the torque values in AP and ML | _____ | Balance was more affected after 24h of SD both in AP and ML directions than after 36h SD. There was no combined effect of SD and vision on balance. |
| Pham et al. 2014 | COPA, CT, and FD | _____ | In foam surface and/or EC, body sway variables derived from the COP increased significantly when TA was larger than 21 h in most subjects. |
| Robillard et al. 2011a | Amplitude, COPV, RMS-amplitude, in AP e ML directions | _____ | SD increased COP-AP range (no cognitive load) condition and decreased COP-ML range and velocity (high cognitive load) |
| Robillard et al. 2011b | Amplitude, COPV in AP e ML directions | _____ | SD increased the AP range of the COP in elderly and young volunteers. Elderly participants had also higher COP speed and more pronounced destabilizing effects of SD with EC. |
| Sargent et al. 2012 | C95 | _____ | For the EC balance task, there was a significant effect of circadian phase such that balance was poorer during the biological night-time than daytime |
| Schlesinger et al. 1998 | RMS - AP | _____ | With IRT, SD increased sway across all postural conditions |
| Sekine and Takahashi 2005 | COPA, Total length of traces | _____ | Total length of COP traces and areas, on stationary platform, increased under sleep restriction |
| Siu et al. 2015 | COPA, total displacement, StDev-AP StDev-ML | _____ | As the net sleep decreased, farmers became less stable: increase in COPA, total displacement, and COP deviations in AP and ML with EO. |
| Smith et al. 2012 | AP and ML COP directions, trace length, C90, COPV | _____ | An effect of extended wake resulted in increased AP sway. Higher values of trace length, C90, ML sway, and COPV, indicating a decreased stability were found with EC. |
| Sobeih et al. 2006 | COPA, length, AP and ML sway | _____ | Lifting task had the greatest impact on postural stability. Sway length increases as firefighters spent more time on duty |
| Tietäväinen et al. 2013 | AP and ML components of the balance signal (CI) | _____ | CI decreased during the 24 h of TA |
| Umemura et al. 2018 | Stability index of COP | _____ | Chronic SD due to social jet-lag affects postural performance on Friday when compared to Monday. |
| Umemura et al. 2019 | TSD, COPA, COPV, StDev-L, StDev-AP, MSA(a-p), MSA(m-l), velocity in AP and ML directions | _____ | Acute sleep deprivation affects postural control, mainly in AP direction and with EC. |
| Uimonen et al. 1994 | BSV, AVEX, AVEY,  MAXX, MAXY | _____ | No balance worsening after 24 h of sleep deprivation (SD) |

ADT: Adaptation test; APSI: antero-posterior stability index; AVEX: vibration-induced shift in the average COP in the lateral direction; AVEY: vibration-induced shift in the average COP in the anterior-posterior direction; AP: anterior-posterior; BSV: length in cm of the path of the COP divided by the time of measurement; C90 and C95: area of the 90% and 95% confidence ellipse enclosing the COP, respectively; CDP: computerized dynamic posturography; CI: complexity index; COM: center of mass; COP: center of pressure; COPA: center of pressure area; COPV: center of pressure velocity; CT: critical time; CTSIB: Clinical Test of Sensory Integration and Balance; EC: eyes closed; EO: eyes open; FD: fractal dimension; FI: Fatigue index ratio between the postural score during 25h study and the average of the four baseline trials FT: feedback time; h: hour; IRT: inhibitor reaction task; L: lateral; LFS: length in function of surface; MAXX: maximum displacement from the average position in the lateral direction; MAXY: maximum displacement from the average position in the anterior-posterior direction; MCFA: most common frequency amplitude; MCSA: most common sway amplitude; MD: mean displacement; MF-AP: medium-frequency-band (0.2-1.0 Hz) power of postural sway in the anterior-posterior direction; MF-L: medium-frequency-band (0.2-1.0 Hz) power of postural sway in the lateral direction; ML: medio-lateral; MLSI: medium-lateral stability index; MTC: motor control test; MSA(a-p): mean sway amplitude in the anterior-posterior; MSA (m-l) mean sway amplitude in the medial-lateral; N: number; NBP: normalized balance platform; OSI: overall stability index; PPC: proprioceptive; PS: postural sway; RA: rectangle area; RI: ratio index [(parameter EC/parameter EO)*100] for these parameters: WPL, UAPL, CA, RA, StDev-L and StDev-AP ; RMS – AP: root mean square of length in the anterior-posterior direction; s: seconds; SCBA: self-contained breathing apparatus; SD: sleep deprivation; SKL: statokinesiogram length; SOT: sensory organization test; SRF: Sway-referenced floor; SRSS: Square root of the sum of the squared differences between adjacent pressure fluctuation signals; SRVS: Sway-referenced visual scene; SS: surface support; StDev: standard deviation; TA: time awake; TOC: time interval for open loop stance control; TSD: total sway displacement; UAPL: unit Area path length; WPL: whole path length of COP; _____: not specified.

**Table 6.** Other variables related to sleep conditions

| Study | Variable | Instrumentation | Protocol | Findings |
| --- | --- | --- | --- | --- |
| Aguiar and Barela 2014 | Subjective sleep quality | PSQI | Before the tests | ________ |
| Albuquerque et al. 2012 | Sleepiness | ESS | Before driving | Circadian and homeostatic influences over balance control performance. Time awake estimation |
| Avni et al. 2006 | Fatigue | SSS and PVT | 12 times (9:00, 13:00, 15:00, 18:00, 21:00, 23:00, 1:00, 3:00, 5:00, 7:00, 9:00, 10:00h) | PVT was correlated with fatigue index; SSS was moderately correlated with it. |
| Cheng et al 2018 | Sleep Quality, sleepiness | SSS, PSQI, | After each postural tests | Higher sleepiness is associated with performance decrease |
| Cuthbertson et al. 2015 | Sleepiness | ESS, SSS, PVT | Before and after the balance tests (SSS and PVT). ESS was used to assess the overall sleepiness | SSS and PVT results worsened with significant increase in score and time. |
| Fabbri et al. 2006 | Alertness, mood and temperature | GVA; digital thermometer | Mood states: tense, happy, sad and calm. Alertness: vigor, tired, effort for everyday tasks and sleepiness. Every 2 hours (22:00-8:00h) | Subjective alertness lowest level at morning, lower than at 22:00h.  Temperature has a similar pattern |
| Furtado et al. 2016 | Sleepiness, subjective sleep quality, chronotype and physical activity | ESS, PSQI, HO, iPAQ | Before the tests | Both groups had similar chronotype and levels of physical activity. |
| Gomez et al. 2008 | Alertness | VAS | 24h and 36h of sleep deprivation (before the posturographic measurements) | Average VAS scores increased at 24 h to 36h SD; negative correlations between VAS scores and hip movement only on 24h of SD in period 3 with EC and in period 1 with EO |
| Haeggstrom et al. 2004 | Alertness | CFF | During sleep deprivation, every 2 hours | Correlation between posturographic parameters and CFF |
| Haeggstrom et al. 2006 | Alertness | CFF | During the sleep deprivation, every 2 hours. | Association between sleep deprivation and CFF. |
| Karita et al. 2006 | Alertness | CFF | __________ | They were not different between the overtime worker groups |
| Liu et al. 2001 | Sleepiness | EEG alpha power and 100 mm VAS | E EEG alpha rhythm recorded with eyes closed for 1 min. | The results suggest that changes in sway during the night are influenced by the increase in sleepiness |
| Ma et al. 2009 | Fatigue | SSS | Before and after a 24-h period of sustained wakefulness | The scores obtained after SD were significantly higher than those recorded at the beginning of the 24-h period of wakefulness |
| Martin et al. 2018 | Chronotype,Oral temperature, Sleepiness, Fatigue | KSS, VAS, thermometer, HO | After the postural tests | Oral temperature, sleepiness, and fatigue showed significant time-of-day variation |
| Morad et al. 2007 | Fatigue | SSS | 9 times (8:00; 9:00; 10:00; 13:00; 1:00; 3:00; 5:00; 8:00; 9:00h) | Significant correlations were found between posturography with EC and SSS excluding the 10:00h point |
| Mori et al. 2017 | CFF, Sleepiness | SSS | Hourly | CFF decreases and SSS increases with SD. Recovery after sleep only in SSS. |
| Nakano et al. 2001 | Alertness and rectal temperature | EEG and thermometer | From 15:00 to 9:00h, hourly | Alertness decreased gradually at night and remained low until morning |
| Narciso et al. 2016 | Sleepiness, attention | KSS and PVT | Before and after the night work | Sleepiness increased; mean reaction time, and number of lapses of attention increased, mean reciprocal reaction time decreased |
| Patel et al. 2008 | Sleepiness | VAS | 24h and 36h of sleep deprivation (before the posturographic measurements) | Score increased at 24 h to 36h SD. Poor correlation with balance control performance indicators. |
| Robillard et al. 2011a | Psychomotor speed | PVT | 25 min before the postural tasks. | Significant correlations between SD effects on the ML range in the EO high cognitive load and the PVT |
| Sargent et al. 2012 | Rectal temperature, sleepiness | Thermistor, KSS | Rectal temperature; KSS (9 times: every 2.5h, starting 1.5h after wake) | Time awake and circadian phase had a significant effect on KSS. KSS increased as prior wake increased, it was higher during biological night than during biological day. |
| Smith et al. 2012 | Arousal and sleepiness | PVT | 18:00-07:00h, hourly | PVT lapses demonstrated a main effect for an increase across 14 h of extended wake. |
| Tietäväinen et al. 2013 | Sleepiness and alertness | SSS; Alertness (alertness = S + C – W) as a function of time since awakening and time of day | Hourly | Alertness was stronger correlated with SSS than CI |
| Umemura et al. 2018 | Chronotype, sleepiness, sleep quality, physical activity | HO, ESS, PSQI, iPAQ | Before tests | Sleep duration desired in HO questionnaire is higher than the total time of sleep in workdays. The ESS is higher in Friday than Monday |

C: Circadian component; CFF: Critical flicker fusion frequency; EC: Eyes closed; EO: Eyes open; ESS: Epworth Sleepiness Scale; HO: Horne and Östberg chronotype morningness–eveningness questionnaire; GVA: Global vigor and affect scale; KSS: Karolinska Sleepiness Scale; PVT: Psychomotor Vigilance Test; S: homeostatic component; PSQI: Pittsburgh Sleep Quality Index; SD: Sleep deprivation; SSS: Stanford Sleepiness Scale; VAS: Visuo-analogue sleepiness scale; W: Sleep inertia components; _____:Not specified.
